# A Japanese version of Mother-to-Infant Bonding Scale: factor structure, longitudinal changes and links with maternal mood during the early postnatal period in Japanese mothers

**DOI:** 10.1007/s00737-012-0291-1

**Published:** 2012-06-26

**Authors:** Keiko Yoshida, Hiroshi Yamashita, Susan Conroy, Maureen Marks, Chianni Kumar

**Affiliations:** 1 Department of Child Psychiatry, Kyushu University Hospital, 3-1-1 Higashi-ku, Fukuoka, Japan; 2 Section of Perinatal Psychiatry, Institute of Psychiatry, University of London, London, UK

**Keywords:** Mother-to-Infant Bonding Scale, Edinburgh Postnatal Depression Scale, Postnatal depression, Factor analysis

## Abstract

The objectives of this study were (1) to develop a Japanese version of Mother-to- Infant Bonding Scale Japanese version (MIBS-J) based on Kumar’s Mother Infant Bonding Questionnaire that could be used to screen the general population for problems in the mother’s feelings towards her new baby and to validate it for clinical use and (2) to examine the factor structure of the items and create subscales of the questionnaire for the Japanese version. The MIBS-J is a simple self-report questionnaire designed to detect the problems in a mother’s feelings towards her newborn baby. Participants (*n* = 554) were recruited at an outpatient clinic of a maternity hospital in a community after 30-weeks gestation. MIBS-J and the Edinburgh Postnatal Depression Scale (EPDS) were administered on the fifth day at the maternity ward and mailed at 1 and 4 months postnatally. Exploratory factor analysis and confirmatory factor analysis demonstrated a two-factor structure out of eight items: lack of affection (LA) and anger/rejection (AR). Chronbach’s *α* coefficients were 0.71 and 0.57, respectively. The LA and AR scores had strong correlations across postnatal times. The mothers with higher (worse) AR scores on the MIBS-J at any of the three periods had higher scores on the EPDS. MIBS-J demonstrated acceptable reliability and reasonable construct validity in this Japanese sample.

## Introduction

Maternal emotional involvement with the baby in the perinatal period has been recognized and named “bonding” and has been mainly discussed in relation to postnatal depression. Although some mothers maintain positive maternal attitudes towards their newborn babies, a proportion of depressed mothers experience severe disorders of affection for their infants (Kumar 1997). Based on interview responses, Brockington et al. (2006a) classified mothers’ attitude towards their babies as: depressed mothers with a normal mother–infant relationship, those with mild mother–infant relationship disorders, and those with pathological anger towards or rejection of the baby. Kumar (1997) also described women’s feelings in the early relationship with their infants, such as absence of affection, hate, rejection, neglect, or impulses to harm. He described this as evidence for a disorder of mother-to-infant bonding. Detailed narrative accounts indicated that such disorders typically would not occur in the absence of a postnatal mental illness, but affected mothers may potentially harm or neglect their babies.

In recent years, research focusing on the mother’s affective behavior towards her newborn baby has increased along with the development of assessment tools. Brockington et al. (2001) developed the Postpartum Bonding Questionnaire (PBQ), which is a self-rating questionnaire. This assessment tool has 25 items for four factors: general factors, rejection and pathological anger, anxiety about the infant, and incipient abuse. Mothers who were referred for emotional or psychological issues in Birmingham and Christchurch participated in a validation study of the PBQ. Satisfactory sensitivity and specificity, particularly for general factor and rejection and pathological anger, was demonstrated.

A preliminary work by Kumar (1997), using items selected from mother’s narrative accounts, resulted in the development of a self-report screening scale, the Mother–Infant Bonding Questionnaire (MIBQ). This scale has nine items: loving, resentful, neutral or felt nothing, possessive, joyful, dislike, protective, disappointed, and aggressive. Taylor et al. (2005) developed a new measurement, the Mother-to-Infant Bonding Scale (MIBS), by adapting Kumar’s MIBQ. They concluded that the questionnaire was acceptable for use with mothers and yielded significant correlations with moods in the early postnatal period. There are studies of test–retest reliability and construct validity for the MIBQ by other researchers (Figueiredo et al. 2005; Taylor et al. 2005; Wittkowski et al. 2007). The MIBQ was later modified by Kumar’s colleague Marks. Compared with the original questionnaire, the wording was changed in three items and the item “scared or panicky” was newly added, resulting in a total of ten items.

Women in Japan become depressed after childbirth with an onset rate similar to European and other countries (Kitamura et al. 2006; Okano et al. 1996; Yamashita et al. 2000; Yoshida et al. 1997). Similarly, Japanese mothers may also have the same problems of mother-to-infant bonding as Western women.

The first aim of the present study was to develop The Japanese version of Mother-to- Infant Bonding Scale (MIBS-J) based on Kumar’s Mother Infant Bonding Questionnaire that could be used to screen the general population for problems in the mother’s feelings towards her new baby and to validate it for clinical use. The second aim of this study was to examine the factor structure of the MIBS items and create subscales of the questionnaire for the Japanese version. Finally, we focused on the impact of the mother’s depression on the emotional involvement with the infant over time.

## Materials and methods

### Participants

All pregnant women who reached 30 weeks of gestation and who were scheduled to give birth at a maternity hospital located in an urban area in Kyushu, Japan were consecutively asked to participate in the present study. The study period was from 30-weeks gestation to 4 postnatal months. The data were collected from May 2000 to August 2001. Out of 892 women who gave written informed consent (only two women did not attend the study), 554 women (62.1 %) completed the study. The mean (SD) age of the mothers was 29.6 (4.3) years. Almost one half of the participants had completed junior college or college. A quarter (24.9 %) of the mothers were employed. All but two of the mothers were married and cohabiting with a partner; 38.7 % were primiparae. All the partners were employed and 39.4 % were manual workers.

### Procedures

**Fig. 1:**
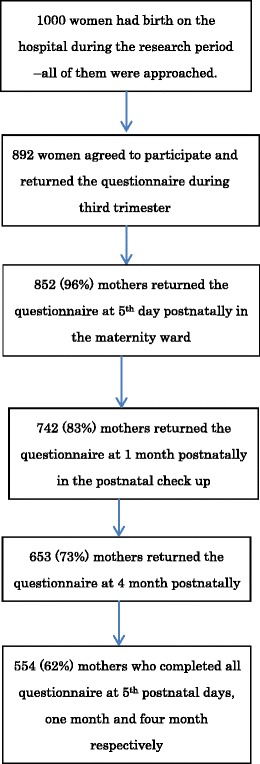
Overview of the mothers’ participation rate

When participants reached 30-weeks gestation, the questionnaires were introduced by a midwife at an outpatient visit at the maternity hospital. The questionnaires included social demographic questions and the Edinburgh Postnatal Depression Scale (EPDS). On the fifth postnatal day, the MIBS-J and the EPDS were distributed and collected by a midwife on a maternity ward (It is typical for the average woman in Japan to stay in the hospital for 4–5 days after giving birth). At 1 and 4 months after delivery, the MIBS-J and the EPDS were sent by post and returned by mail. Numbers of respondents were 860, 759 and 658 at the three postnatal assessment points, respectively. Because we were interested in the linkage of data between these three assessment occasions, we selected 554 women with complete information for further analysis (Fig. 1).

### Measures

#### Mother-to-Infant Bonding Scale

The MIBS is a self-report scale composed of ten items on a four-point Likert scale (from 0, “not at all” to 3, “very much”). Some MIBS items are reversed. A high score indicates worse mother to infant bonding. We translated the MIBS into Japanese, and then our Japanese version was retranslated back into English by a native English translator who was unaware of the original wording to confirm that the translation was consistent with the original meaning.

#### Edinburgh Postnatal Depression Scale

The EPDS is a self-report questionnaire composed of ten items scored on a four-point Likert scale (0–3) designed to assess pregnancy and postpartum depression Cox et al. (1987). Numerous studies have used this instrument during the antenatal and/or postpartum period. The EPDS Japanese version showed good internal consistency (Cronbach alpha = 0.78) and test–retest reliability (Spearman correlation = 0.92) (Okano et al. 1996). A score equal to or higher than 9 was designated to screen for minor and major depressive episodes, with a sensitivity of 75 and 82 % with specificity of 93 and 95 %, respectively (Okano et al. 1996; Yamashita et al. 2000).

#### Sociodemographic items

The self-report questionnaire was administered at 30-weeks gestation and included socio-demographic and medical items as follows: maternal age, educational level, occupational status, marital status, cohabiting status, partner’s occupational status, obstetric history, and psychiatric history.

### Statistical analysis

We calculated descriptive statistics for the ten items of MIBS as shown in Table 1. All of the items were positively skewed (skew values greater than 1) in all of different timings. Based on these results, we found violation of the normality assumption for the items and substantive transformation were necessary for the subsequent factor analysis (Tabachnick and Fidell 2007). For our current data, we found a log transformation was the most effective transformation. For all the analysis but exploratory and confirmatory factor analysis, we used raw score of MIBS-J taking account of convenience for clinical use.

**Table 1:** Means and SDs of the MIBS-J items at day 5, 1 month, and 4 months after childbirth in half of the sample (*n* = 554)

Item no	MIBS-J items	Day 5		Month 1		Month 4	
		M (SD)	Skewness	M (SD)	Skewness	M (SD)	Skewness
1	Feel loving towards my baby	0.12 (0.36)	3.07	0.12 (0.34)	2.65	0.07 (0.26)	3.36
2	Feel scared or panicky when I have to do something for my baby	0.64 (0.67)	0.65	0.45 (0.56)	0.73	0.28 (0.50)	1.73
3	Feel resentful towards my baby	0.06 (0.27)	4.65	0.17 (0.40)	2.10	0.20 (0.40)	1.69
4	Feel nothing towards my baby	0.04 (0.27)	7.53	0.03 (0.22)	10.74	0.02 (0.14)	7.14
5	Feel angry with my baby	0.03 (0.18)	7.72	0.08 (0.28)	3.63	0.09 (0.31)	3.61
6	Enjoy doing things with my baby	0.36 (0.59)	1.67	0.39 (0.56)	1.09	0.28 (0.52)	1.71
7	Wish my baby was different	0.09 (0.38)	5.71	0.06 (0.27)	4.85	0.07 (0.32)	4.57
8	Feel protective towards my baby	0.11 (0.38)	4.01	0.05 (0.22)	4.04	0.09 (0.39)	5.17
9	Wish I did not have my baby	0.04 (0.21)	6.09	0.06 (0.23)	3.88	0.09 (0.35)	4.68
10	Feel close to my baby	0.17 (0.41)	2.40	0.07 (0.28)	3.77	0.06 (0.30)	6.49

After randomly dividing the subjects into two groups, we conducted an exploratory factor analysis (EFA) of the ten items of MIBS-J using data from one group of mothers (group 1; *n* = 267). The MIBS-J scores over the three occasions (day 5, 1 month, and 4 months) were combined for EFA so that 801 cases were analyzed. Because all factors were considered dependent upon each other, the factor solution was sought after Promax rotation, which is diagonal (oblique) rotation. The number of factors was determined by scree test (Cattel 1966). In order to create a subscale of the MIBS-J, we extracted items for each subscale if they were loaded greater than 0.4 on a particular factor but less than 0.3 on other factors.

The factor structure derived from the EFA was confirmed by a confirmatory factor analysis (CFA) in the other half of the group (group 2; *n* = 861, i.e. 287 × 3). The fit of each model with the data was examined in terms of chi-squared (CMIN), goodness-of-fit index, adjusted goodness-of-fit index, comparative fit index (CFI), root mean square error of approximation (RMSEA) and root mean square residual (RMR).

According to conventional criteria, a good fit would be indicated by CMIN/*df* < 2, CFI > 0.97, RMSEA < 0.08, and RMR < 0.08 (Schermelleh-Engell et al. 2003). The Akaike Information Criterion (AIC) was used to compare different models; a model with an AIC of at least 2 points lower is regarded as a better model.

Alphas for the two hypothesized subscales were calculated to examine the internal reliability of the MIBS-J.

We also constructed a correlation matrix with the EPDS. This analysis provided evidence of predictive and concurrent validity.

The significance of differences between MIBS-J subscale ratings at the three occasions was determined by one-way ANOVA with Bonferroni post hoc comparison. All the statistical analyses were conducted using the Statistical Package for Social Science (SPSS) version 20.0 and Amos 20.0.

## Results

### Descriptive statistics

The mean and SDs of all the MIBS-J items at day 5, 1 month, and 4 months postnatally are shown in Table 1. All scores were relatively low.

### Factor analysis of MIBS-J items

All the log-transformed items of the MIBS-J were entered into an EFA. This yielded two factors (Table 2). The first factor was loaded by four items: “I feel protective towards my baby” (reverse item); “I feel close to my baby” (reverse item); and “I feel loving towards my baby” (reverse item). These items reflected maternal lack of positive affection and intimacy towards the baby. We named this factor Lack of Affection (LA). The second factor was loaded by three items: “I feel scared or panicky when I have to do something for my baby”; “I feel angry with my baby”; “I feel resentful towards my baby”; and “I wish my baby was different”. These items reflected mothers’ anger and rejection towards the baby. We named this factor anger and rejection (AR).

**Table 2:** Factor structure of the MIBS-J items over the three occasions (Day 5, 1 month, and 4 months after the childbirth) in half of the sample (group 1; *n* = 801)

Item no	MIBS-J items	Factor 1	Factor 2
8	Feel protective towards my baby	**0.83**	−0.13
10	Feel close to my baby	**0.81**	−0.06
1	Feel loving towards my baby	**0.74**	0.03
6	Enjoy doing things with my baby	**0.62**	0.10
5	Feel angry with my baby	−0.11	**0.79**
3	Feel resentful towards my baby	−0.06	**0.78**
7	Wish my baby was different	−0.00	**0.54**
2	Feel scared or panicky when I have to do something for my baby	0.00	**0.47**
9	Wish I did not have my baby	0.36	0.43
4	Feel nothing towards my baby	0.24	0.39
	% Variance explained	31.9 %	14.2 %

Bolded values are factor loadings with >0.4 on one factor and <0.3 on another factor.

**Fig. 2:**
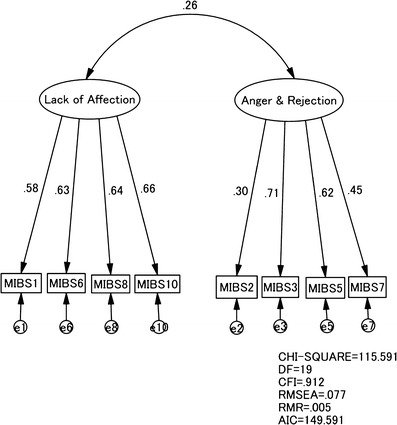
Confirmatory factor analysis of the MIBS-J items over the three occasions (day 5, 1 month, and 4 months after childbirth) in half of the sample (group 2; *n* = 861, i.e. 287 × 3)

CFAs were conducted to test the two factor model identified in the EFA of the current study using the combined data of three occasions of Group 2. The model derived from the EFA showed good fit with the data CMIN/*df* = 6.08 CFI = 0.912, RMSEA = 0.077, and RMR = 0.005 (Fig. 2). The two latent factors were moderately correlated with each other. We also tested the one-factor model according to Taylor et al. (2005); that model did not show goodness of fit with our data (CMIN/*df* = 12.8, CFI = 0.499, RMSEA = 0.203, RMR = 0.007, AIC = 207.127).

In order to examine stability of the factor structure of the MIBS-J, we conducted a series of CFAs using all the occasions from group 2. The path values and accompanying fit indices are shown in Table 3. For all the occasions, normed chi-square (NC) (*χ* ^2^/*df*) values were <5, indicating a reasonable model of fit (Bollen 1989; Kline 1993). Examination of CFI and RMSEA values revealed the current model to meet approximate fit criteria only at 5 days postnatally. The fitness of the model to the data was acceptable for the data on day 5, but worse for the data on months 1 and 4.

**Table 3:** Confirmatory factor analysis of the MIBS-J at day 5, 1 month, and 4 months after the childbirth in the other half of the sample (group 2; *n* = 287)

Item no	MIBS-J items	Day 5	Month 1	Month 4
	Lack of affection (LA)			
8	Feel protective towards my baby	0.53	0.76	0.66
10	Feel close to my baby	0.66	0.73	0.67
1	Feel loving towards my baby	0.57	0.59	0.54
6	Enjoy doing things with my baby	0.62	0.61	0.60
	Anger and rejection (AR)			
5	Feel angry with my baby	0.60	0.61	0.64
3	Feel resentful towards my baby	0.72	0.69	0.72
7	Wish my baby was different	0.55	0.29	0.55
2	Feel scared or panicky when I have to do something for my baby	0.09	0.59	0.36
	Covariance between lack of affection and anger/rejection	0.43	0.14	0.21
	Chi-squared/*df*	2.37	3.73	2.96
	CFI	0.922	0.889	0.905
	RMSEA	0.069	0.098	0.083
	RMR	0.005	0.007	0.004
	AIC	78.99	104.97	90.22

### Internal consistency of Mother-to-Infant Bonding Scale

As for the combined data of group 1, internal consistency (Cronbach’s alpha) of the four items belonging to the lack of affection (LA) subscale was 0.71, while that of the three items belonging to the Anger and Rejection (AR) subscales was 0.51, showing reasonable internal consistency.

### Construct validity of Mother-to-Infant Bonding Scale-J

The EPDS scores were positively correlated with both LA and AR subscales at the three occasions. Thus negative affect of mothers correlated with poorer bonding. The strongest correlations were between Anger and Rejection and EPDS, especially at 1 month postpartum.

### Test–retest reliability and longitudinal change of MIBS-J

All MIBS-J subscales were correlated significantly with day 5, 1 month, and 4 months among a total of 554 mothers (Table 4). Bonding scores at day 5 correlated significantly with scores at 1 month and 4 months, demonstrating the stability of the MIBS-J over the period of the current study.

**Table 4:** Pearson’s correlations of MIBS-J-subscale scores and the EPDS, *n* = 554

MIBS-J	1	2	3	4	5	6	EPDS Day 5	EPDS 1 month	EPDS 4 months
LA day 5		0.235**	0.460**	0.182**	0.336**	0.171**	0.181**		
AR day 5			0.137*	0.455**	0.136**	0.335**	0.360**		
LA 1 month				0.239**	0.439**	0.188**		0.224**	
AR 1 month					0.139**	0.530**		0.514**	
LA 4 months						0.253**			0.199**
AR 4 months									0.392**

*p < 0.05; **p < 0.01

ANOVA was used to compare the distributions of subscales. Results showed average LA scores were significantly lower at 4 months than those recorded at 5 days and 1 month. This indicated that loss of affection had improved considerably between 1 and 4 months (*F*(2,1106) = 14.6, *p* < 0.01). AR scores were significantly lower at 4 months compared to scores at day 5. This indicated that feelings of anger and rejection had decreased considerably between day 5 and 4 months (*F* (2, 1,106) = 9.6, *p* < 0.01) (Table 5).

**Table 5:** Mean of MIBS-J subscale scores at day 5, 1 month and 4 months postpartum, *n* = 554

	Mean (SD)	*F* score
LA Day 5	0.84 (1.41)	14.6*
LA 1 month	0.79 (1.33)	
LA 4 months	0.53 (1.15)	
AR Day 5	0.84 (0.99)	9.6*
AR 1 month	0.81 (1.16)	
AR 4 months	0.65 (1.08)	

One way ANOVA

*p < 0.01

## Discussion

### Psychometric properties of Japanese Mother-to-Infant Bonding Scale

The response rate for this prospective study was good; 892 women were recruited during pregnancy and 554 returned fully completed questionnaires for all items at three time points. The questionnaire was acceptable to the mothers. In comparison with the distribution of the eight-item MIBS score of mothers in England (Taylor et al. 2005), the seven-item MIBS-J score for Japanese mothers showed a similar skewed pattern (the median and range) at any time point. Both samples were derived from average maternity hospitals in a community where participants were likely to have similar socio-demographic backgrounds. Therefore, it is suggested that the MIBS-J may be better at detecting more abnormal responses, rather than subtle differences within a normal population as in England. However, with the use a self-report questionnaire, we should be aware of Japanese mothers’ “modest” expressions regarding their feelings. For example, a cut-off point of the EPDS with a Japanese sample is 8/9 rather than 12/13 (Okano et al. 1996; Yamashita et al. 2000).

### Factor structure of Mother-to-Infant Bonding Scale

The present study suggested that the MIBS-J items fit the two-factor model for data in EFA. The psychometric properties of the MIBS were investigated in several studies (Figueiredo et al. 2005; Taylor et al. 2005; Wittkowski et al. 2007). It was proposed that the original nine-item MIBS score should be recalculated as a single-factor scale after discarding one item (possessive). However based on the model of the current study, the questions on the MIBS-J were rescored using eight items divided into two subscales resulting in a better internal consistency than a single-factor model.

The strength of this study is that we conducted an EFA for half of the participants and confirmed stability of the factor structure in the other half using CFA to compare our three time points. Results of a CFA in the current study confirmed the scale’s multidimensionality. The identified two-factor model had an adequate fit to the data and was superior in statistical significance to Taylor et al.’s (2005) unidimensional model. According to the two-factor model, it is likely to detect a failure of positive bonding (Lack of affection) and anger/rejection towards the baby. These subscales correspond with “Impaired bonding” and “Rejection and Anger” in the 25-item Postpartum Bonding Questionnaire by Brockington et al. (2001) that revealed a four-factor structure. Wittkowski et al. (2010) used the PBQ in a mother baby unit with psychiatric inpatient treatment; they proposed that the three factor model (omitting “risk of abuse” from the original four factor model) was the most stable and clinically meaningful solution based on the research.

### The course of MIBS-J

Two subscale scores of MIBS-J indicated different transition patterns over time after delivery. Lack of affection score significantly decreased from day 5 to 4 months postnatally. This finding is in accordance with the notion that a considerable proportion of mothers have a delay (3 months postnatal) in the onset of maternal affection; however, very few mothers are indifferent towards their baby by 6 months (Robson and Kumar 1980).

Similarly, rejection and anger subscale scores were inclined to decrease throughout the course of the research period. Maternal anger is associated with child rearing stress, such as having a “difficult” baby. Maternal aggression or neglect towards infants might be mainly linked to severe postpartum depression (Reck et al. 2004). Mothers in stressful parenting situations, such as difficulties of breast-feeding, infant colic or prolonged crying, might become gradually depressed and experience increased negativity towards the baby (Vik et al. 2009).

### Association with maternal mental health

Mothers with greater depressive symptoms reported lower feelings of bonding with their infants as shown in the correlation between the EPDS and anger and rejection subscale. However, since correlations between the EPDS and lack of affection subscale at each time point were not significant, this suggests that the loss of positive feelings towards the infant is not just an inherent part of the postpartum depressive syndrome but an independent and specific phenomenon. In light of changes in maternal depressive symptoms over time, a substantial proportion became depressed at the 6- or 9-month interval (Ammerman et al. 2009; Howell et al. 2009). If the change of depressive symptoms was associated with change in parenting problems (Waylen and Stewart-Brown 2010), the increase of scores in the rejection and anger subscale is likely to reflect parenting difficulties arising from depressive symptoms.

### Limitations

Several limitations of this study need to be addressed. First, the present sample is not representative of the total population because of the relatively high educational level and economical status. A clinical population or high risk group who receive home visits may present with different features. Secondly, as for the validation procedure, this study was based on a self-report questionnaire without diagnostic procedures based on external criteria, such as the Birmingham Interview for Maternal Mental Health (Brockington et al. 2006b). We did not use other questionnaires which detect disorders in the early emotional bond between a mother and her newborn infant; therefore, convergent validity was not investigated in this study. Thirdly, no data about the infants were collected in this study. The bonding process in infancy is influenced by the characteristics and behaviour of the infant.

### Clinical implications

As shown in previous studies in western countries, there is a general trend towards better bonding in the first 4 months postpartum among Japanese mothers. Longitudinal changes in the lack of affection subscale indicates that maternal bonding develops progressively over the first 12 weeks. It is important to note that the EPDS showed the greatest correlation with anger and rejection. This suggests that depressive symptoms are directly linked with worse bonding and especially anger and rejection might be linked with a risk of maltreatment or child abuse.

## Appendix

### Mother-to-Infant Bonding Scale

I would like to know how you have been feeling about your baby lately. Listed below are some of the feelings mothers have about their babies. Please underline the answer which comes closest to how you usually feel about your baby, not just how you feel today. Please complete ALL items.

## References

[CR1] Ammerman R, Putnam F, Altaye M, Chen L, Holieb L, Stevens J, Short J, Van Ginkel J (2009). Changes in depressive symptoms in first time mothers in home visitation. Child Abus Negl.

[CR2] Bollen KA (1989) Structual equation with latent variables. In: Wiley Series in Probability and mathematical statistics. Wiley, New York

[CR3] Brockington IF, Aucamp HM, Fraser C (2006). Severe disorders of the mother–infant relationship: definitions and frequency. Arch Womens Ment Health.

[CR4] Brockington IF, Fraser C, Wilson D (2006). The Postpartum Bonding Questionnaire: a validation. Arch Womens Ment Health.

[CR5] Brockington IF, Oates J, George S, Turner D, Vostanis P, Sullivan M, Loh C, Murdoch C (2001). A screening questionnaire for mother–infant bonding disorder. Arch Womens Ment Health.

[CR6] Cattel R (1966). The scree test of the number of factors. Multivariate Behav Res.

[CR7] Cox J, Holden J, Sagovsky R (1987). Detection of postnatal depression. Development of the 10-item Edinburgh Postnatal Depression Scale. Br J Psychiatry.

[CR8] Figueiredo B, Marques A, Raquel C, Alexandra P, Alvaro P (2005). Bonding: escala para avaliar o envolvimento emocional dos pais com o bebé. Psychologica.

[CR9] Howell E, Mora P, DiBonaventura M, Levethal H (2009). Modifiable factors associated with changes in postpartum depressive symptoms. Arch Womens Ment Health.

[CR10] Kitamura T, Yoshida K, Kinoshita K, Hayashi M, Toyoda N, Ito M, Kudo N, Tada K, Kanazawa K, Sakumoto K, Satoh S, Furukawa T, Nakano H (2006). Multicentre prospective study of perinatal depression in Japan: incidence and correlates of antenatal and postnatal depression. Arch Womens Ment Health.

[CR11] Kline P (1993). Handbook of psychological testing.

[CR12] Kumar R (1997). “Anybody’s child”: severe disorders of mother-to-infant bonding. Br J Psychiatry.

[CR13] Okano T, Murata M, Masuji F, Tamaki R, Nomura J, Miyaoka H, Kitamura T (1996). Validation and reliability of Japanese version of EPDS (Edinburgh Postnatal Depression Scale). Arch Psychiatr Diagn Clin Eval.

[CR14] Reck C, Hunt A, Fuchs T, Weiss R, Noon A, Möhler E, Downing G, Tronick E, Mundt C (2004). Interactive regulation of affect in postpartum depressed mothers and their infants: an overview. Psychopathol.

[CR15] Robson K, Kumar R (1980). Delayed onset of maternal affection after childbirth. Br J Psychiatry.

[CR16] Schermelleh-Engell K, Moosbrugger H, Müller H (2003). Evaluating the fit of structural equation models: tests of significance and desctiptive goodness-of-fit measures. Methods Psychol Res Online.

[CR17] Tabachnick BC, Fidell LS (2007). Using multivariate statistics.

[CR18] Taylor A, Atkins R, Kumar R, Adams D, Glover V (2005). A new Mother-to-Infant Bonding Scale: links with early maternal mood. Arch Womens Ment Health.

[CR19] Vik T, Grote V, Escribano J, Socha J, Verduci E, Fritsch M, Carlier C, von Kries R, Koletzko B, Group ECO (2009). Infantile colic, prolonged crying and maternal postnatal depression. Acta Paediatr.

[CR20] Waylen A, Stewart-Brown S (2010). Factors influencing parenting in early childhood: a prospective longitudinal study focusing on change. Child Care Health Dev.

[CR21] Wittkowski A, Wieck A, Mann S (2007). An evaluation of two bonding questionnaires: a comparison of the Mother-to-Infant Bonding Scale with the Postpartum Bonding Questionnaire in a sample of primiparaous mothers. Arch Womens Ment Health.

[CR22] Wittkowski A, Williams J, Wieck A (2010). An examination of the psychometric properties and factor structure of the Post-partum Bonding Questionnaire in a clinical inpatient sample. Br J Clin Psychol.

[CR23] Yamashita H, Yoshida K, Nakano H, Tashiro N (2000). Postnatal depression in Japanese women. Detecting the early onset of postnatal depression by closely motintoring the postpartum mood. J Affect Disord.

[CR24] Yoshida K, Marks M, Kibe N, Kumar R, Nakano H, Tashiro N (1997). Postnatal depression in Japanese women who have given birth in England. J Affect Disord.

